# In silico investigation into dendritic cell regulation of CD8Treg mediated killing of Th1 cells in murine experimental autoimmune encephalomyelitis

**DOI:** 10.1186/1471-2105-14-S6-S9

**Published:** 2013-04-17

**Authors:** Richard A Williams, Richard Greaves, Mark Read, Jon Timmis, Paul S Andrews, Vipin Kumar

**Affiliations:** 1 Department of Computer Science, University of York, York, YO10 5GH, UK; 2 Department of Electronics, University of York, York, YO10 5DD, UK; 3 Laboratory of Autoimmunity, Torrey Pines Institute for Molecular Studies, San Diego, CA 92121-1122, USA

## Abstract

**Background:**

Experimental autoimmune encephalomyelitis has been used extensively as an animal model of T cell mediated autoimmunity. A down-regulatory pathway through which encephalitogenic CD4Th1 cells are killed by CD8 regulatory T cells (Treg) has recently been proposed. With the CD8Treg cells being primed by dendritic cells, regulation of recovery may be occuring around these antigen presenting cells. CD4Treg cells provide critical *help *within this process, by licensing dendritic cells to prime CD8Treg cells, however the spatial and temporal aspects of this *help *in the CTL response is currently unclear.

**Results:**

We have previously developed a simulator of experimental autoimmune encephalomyelitis (ARTIMMUS). We use ARTIMMUS to perform novel *in silico *experimentation regarding the priming of CD8Treg cells by dendritic cells, and the resulting CD8Treg mediated killing of encephalitogenic CD4Th1 cells. Simulations using dendritic cells that present antigenic peptides in a mutually exclusive manner (either MBP or TCR-derived, but not both) suggest that there is no significant reliance on dendritic cells that can prime both encephalitogenic CD4Th1 and Treg cells. Further, *in silico *experimentation suggests that dynamics of CD8Treg priming are significantly influenced through their spatial competition with CD4Treg cells and through the timing of Qa-1 expression by dendritic cells.

**Conclusion:**

There is no requirement for the encephalitogenic CD4Th1 cells and cytotoxic CD8Treg cells to be primed by the same dendritic cells. We conjecture that no significant portion of CD4Th1 regulation by Qa-1 restricted CD8Treg cells occurs around individual dendritic cells, and as such, that CD8Treg mediated killing of CD4Th1 cells occurring around dendritic cells is not critical for recovery from the murine autoimmune disease. Furthermore, the timing of the CD4Treg licensing of dendritic cells and the spatial competition between CD4Treg and CD8Treg cells around the dendritic cell is critical for the size of the cytotoxic T lymphocyte response, because dendritic cells have a limited lifespan. If treatments can be found to either speed up the licensing process, or increase the spatial competitiveness of CD8Treg cells, the magnitude of the cytotoxic T lymphocyte response can be increased.

## Background

**Figure 1:**
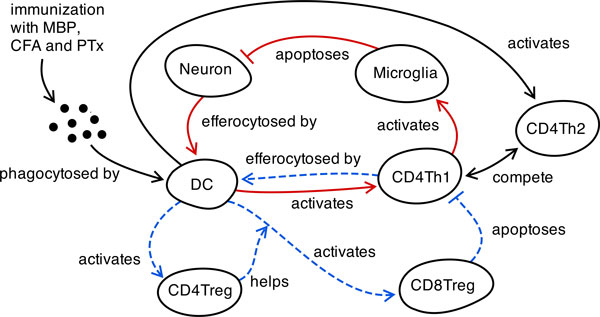
**Cell network model of CD8Treg mediated regulation within the ARTIMMUS EAE simulator**. Computer simulations commence following immunization with MBP, which DCs subsequently engulf and present on MHC class II molecules to facilitate activation of MBP-specific encephalitogenic CD4Th1 cells. Effector CD4Th1 cells migrate to the CNS and initiate the autoimmune pathway (red solid arrows) through microglia induced killing of neurons (our computational abstraction of demyelination within EAE). Effector CD4Th1 cells undergo activation-induced cell death at the end of the cell lifecycle. Apoptotic CD4Th1 cells migrate out of the CNS, and are efferocytosed by CLN and spleen-resident DCs. They may also be efferocytosed in the CNS by DCs that migrate to the CLN upon maturation. These DCs present TCR peptides, which initiate the down-regulatory pathway (blue dashed arrows) through activation of CD4Treg cells and CD8Treg cells [5], and subsequent killing of the encephalitogenic CD4Th1 cells. On a population level, regulation of CD4Th1 cells leads to a type2 deviation [23], resulting in down-regulation of the autoimmune response. Adapted from [20].

Under normal circumstances, self-tolerance mechanisms ensure the host's immune system does not react against *self *antigens [1]. When these self-tolerance mechanisms fail, autoimmune responses occur, which may culminate in the development of autoimmune disease(s). Experimental autoimmune encephalomyelitis (EAE) is an animal model of T cell mediated autoimmune diseases in general, and multiple sclerosis (MS) in particular [2]. The animal disease is mediated through a network of cells (see Figure 1); encephalitogenic T helper cells are activated in the peripheral lymph nodes following immunization for EAE, and migrate to the central nervous system (CNS) where they induce activation of microglia, macrophages and dendritic cells (DCs) [3]. The resultant inflammation causes demyelination of neurons, prompting the presentation of myelin basic protein (MBP) to additional encephalitogenic T cell populations in the cervical lymph nodes (CLN) by migratory DCs. The spontaneous recovery that occurs following autoimmune episodes is associated with a major reduction in the T cell infiltrate in the CNS [4]. Recent evidence indicates that encephalitogenic CD4Th1 cells undergo selective T cell mediated regulation; CD4 T regulatory cells (CD4Treg) *help *DCs in priming CD8 T regulatory cells (CD8Treg) that induce selective apoptotic elimination of CD4Th1 cells [5,6]. The DCs mediate down-regulation of the autoimmune response through the expression of Qa-1 (a MHC class Ib molecule, first discovered by Cantor et al [6]), which facilitates the priming of CD8Treg cells, for subsequent killing of encephalitogenic CD4Th1 cells [7].

The exact location of killing of the encephalitogenic CD4Th1 cells, and thus down-regulation of the autoimmune response by CD8Treg cells, is unknown. One possibility is that DCs migrating from the CNS, potentially carrying both MBP (from neurons) and T cell receptor (TCR) derived peptides, are simultaneously priming both CD4Th1 and Treg cell populations [8], and that regulation of autoimmunity is taking place around the DC. Effector CD4Th1 cells contain framework region 3 (Fr3) and complementarity determining region 1/2 (CDR1/2) peptides within their TCR molecules, and once efferocytosed by a DC, there is a probabilistic chance that the DC will present the Fr3 and CDR1/2 peptides on MHC class II molecules. Tang et al [9] suggest that the CD8Treg response is cross-primed by DCs that have efferocytosed apoptotic CD4Th1 cells and present TCR-derived peptide to both CD4Treg and CD8Treg cells [8,10]. Kumar and colleagues [11-13] found that CD4Treg cells may then become activated by binding to the Fr3-MHCII complex of an antigen presenting cell (e.g. DC or microglia). Conversely, CD8Treg cells recognise TCR-derived peptides relating to the CDR1/2 regions, which have been cross-presented on MHC class I molecules.

**Figure 2:**
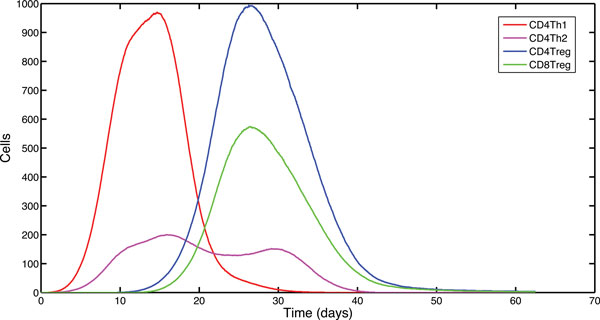
**Control dynamics of the ARTIMMUS simulator**. Median population curves for effector CD4Th1, CD4Th2, CD4Treg and CD8Treg cells as total numbers across all spatial compartments. CD4Th1 cells rapidly proliferate approximately 3-4 days following immunization for EAE, peaking at approximately day 12. The population of CD4Treg cells begin to proliferate shortly before the peak in CD4Th1 cells is achieved. Due to their *helper *function on CD8Treg cells, these show a similar proliferative timecourse, although having smaller total numbers. Although the CD4Th2 cells compete with CD4Th1 cells and contribute to down-regulation of the autoimmune response, their total numbers are significantly lower than the encephalitogenic CD4Th1 cells. The majority of CD4Treg and CD8Treg cells are primed within the spleen (data not presented), and there are approximately 66% more CD4Treg than CD8Treg cells. The autoimmune response is complete and returns to a *normal *state shortly after day 60 (after Read [14]).

The spatial and temporal features of EAE lend themselves well to being modelled using an agent-based approach. In this paper we investigate the effect of regulation of CD4Th1 cells by CD8Treg cells through use of a simulation (ARTIMMUS), which we have previously developed to support investigation into EAE [14]. We investigate three hypotheses, the first hypothesis is that a significant portion of CD4Th1 and CD8Treg cell priming occurs simultaneously around the same DCs. The second hypothesis (termed the *age of licensing *hypothesis) is that the timing of Qa-1 expression by DCs has a significant effect on the size of the CD8Treg effector cell population. The third hypothesis (termed the *spatial saturation *hypothesis) is that spatial competition between CD4Treg and CD8Treg cells around DCs on which they prime, has a significant effect on the size of the CD8Treg effector cell population. We have performed a number of *in silico *experiments to further understand the priming dynamics of CD8Treg cells by DCs against these three hypotheses. In order to establish control simulation dynamics, we have reproduced the baseline generated from development of the simulator [14]. Figure 2 illustrates the relevant cell populations within the simulator under *control *conditions.

Although much is known on the mechanics of antigen-presentation [15], it is unknown whether the individual DC can simultaneously prime both encephalitogenic CD4Th1 and Treg cell populations. If so, then key regulatory action might be taking place around the DCs that prime these populations. The first experiment therefore examined priming dynamics of CD8Treg cells in relation to the antigenic peptide presentation by DCs to ascertain whether an individual DC can prime both encephalitogenic CD4Th1 and CD8Treg cells in parallel. The second and third experiments investigate the manner that *help *(as provided by CD4Treg cells) can modulate the regulatory immune response. The time required for CD4Treg to license the DC for Qa-1 expression, and spatial competition between CD4Treg and CD8Treg priming on the same DC, may in fact have a limiting effect on the magnitude of the CD8Treg cytotoxic T lymphocyte (CTL) response. In the control simulation, it takes approximetaly 60 hours for CD4Treg cells to mature into effectors and license DCs for Qa-1 expression. Manipulation of the time at which DCs express Qa-1 may affect the timing of CD8Treg cell priming and activation, with earlier expression of Qa-1, permitting populations of CD8Treg cells more time for proliferative activity within the lifetime of DCs on which they prime. Similarly, manipulation of the spatial competition between CD4Treg and CD8Treg cells for binding space around DCs, may also affect CD8Treg dynamics. Both CD4Treg and CD8Treg cells can prime on the same DCs, and therefore compete for spatial access to DC. Since CD4Treg cells may begin priming immediately upon DC maturation, with CD8Treg cells waiting for the DC to be licensed, CD4Treg cells would be expected to occupy a greater fraction of the total space around the DC. If spatial competition is a limiting factor in CD8Treg population expansion, then an increase in the competitive strength shown by CD8Treg cells, would enhance the resulting CTL response, as a greater number of CD8Treg cells would be primed.

## Results and discussion

### Effective regulation does not require CD4Th1 and CD8Treg to prime on the same DC

We examined the peptide presentation profiles for DCs *in silico *using ARTIMMUS, and showed that the vast majority (circa 90%) do not present antigenic peptides, and of those that do, the overwhelming majority (circa 90%; or 9% of total DCs) present either MBP or TCR-derived peptides, but not both. In fact, only 1% of DCs within the simulation present both MBP and TCR-derived peptides.

**Figure 3:**
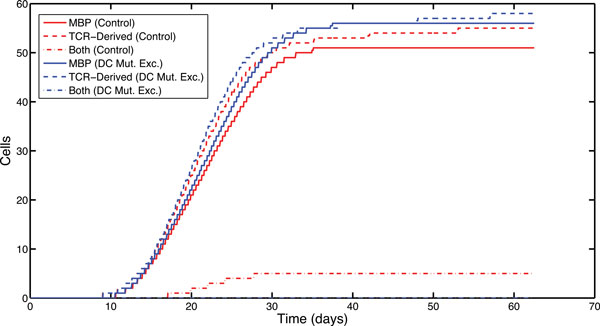
**Mutually exclusive antigenic presentation by DCs**. Median DC populations in a sample of 1,000 simulator runs. The graph shows DC populations for control and *mutually exclusive *presentation experiments. Inspection of data (not shown) confirmed that *mutually exclusive *DC simulation experiments resulted in no DCs presenting both MBP and TCR-derived peptides. A-Test scores showed a small effect size difference in TCR-derived expression (score = 0.427) and MBP presentation (score = 0.635), although no real scientific significance, and virtually no effect size difference in total DCs (score = 0.503) or cells presenting no antigen (score = 0.478).

When imposing mutually exclusive presentation on DCs (Figure 3), where a DC cannot simultaneously present both MBP- and TCR-derived peptides, we find a small reduction (A-Test score = 0.427) in the number of DCs presenting TCR-derived peptides, and a small increase in the number of DCs presenting MBP-derived peptides (A-Test score = 0.635). This leads to an approximate 10% reduction in the priming of CD8Treg cells within the system, which leads to an approximate 5% reduction overall in the total number of CD4Th1 cells which are killed by the CD8Treg population. Next we examined the effect of mutually exclusive presentation on priming DCs within the cervical lymph node (CLN) where DCs prime both populations of T cells (CD4Th1 and CD4Treg). We find a small, but not scientifically significant, reduction in the CD4Th1 killing facilitated by CD8Treg cells with respect to control simulations (A-Test score for total CD4Th1 apoptosis = 0.415, and CD4Th1 apoptosis within the CLN = 0.44). This indicates that enforcing mutually exclusive antigenic presentation has no scientifically significant effect on the regulating capacity, and as such, we conjecture that killing does not take place around the DC.

### Timing of Qa-1 expression by dendritic cells modulates the regulatory response

The control simulation uses two-way signalling between DCs and CD4Treg cells. CD4Treg cells are activated through a MHCII-Fr3 complex (signal 1), and once they become effector cells, if a CD40-CD40L bind is also formed (signal 2), the CD4Treg *licenses *the DC to express Qa-1 (we have termed this *help *within the abstracted model represented by the simulator). Expression of Qa-1 leads to the activation of CD8Treg cells (through cross-priming by a Qa-1-CDR1/2 complex), and subsequent apoptosis of CD4Th1 cells. This raises the question of how variations in the timing of CD40-CD40L interactions may modulate the CD8Treg CTL response.

**Figure 4:**
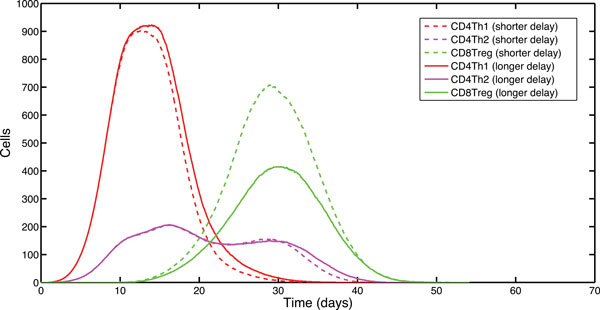
**Effect of the timing of Qa-1 expression on CD8Treg cell population**. Median T cell effector populations in a sample of 1,000 simulator runs. The graph shows T cell effector populations with delayed Qa-1 expression on DC. The dashed curves reflect Qa-1 expression using the shorter delay (60 hours), whilst the solid curves reflect Qa-1 expression using the longer delay (82 hours). There are no curves for the CD4Treg populations as CD4Treg were abrogated in the simulation to facilitate the specific timing of Qa-1 expression by DCs.

The limiting nature of time required for DC licensing on the CD8Treg response was investigated through delaying the time upon which a DC can express Qa-1, and thus gain the ability to prime CD8Treg cells (Figure 4, see methods section for the derivation of this figure). In this context, *timing of Qa-1 expression*, means the expression of optimal levels of Qa-1/TCR-peptide complexes (along with costimulatory molecules) on the surface of DCs enabling the priming of the CD8Treg. This Qa-1 expression delay has been defined within the simulation as the median time taken for a DC to become licensed by a CD4Treg under control conditions (60 hours). We removed the CD4Treg population from our experimental simulations to allow manipulation of the time delay before Qa-1 is constitutively expressed, and upregulate Qa-1 expression on DCs after a pre-specified period of time following their maturation. We demonstrate a medium-level increase to the peak population of CD8Treg cells (relative to the control dynamics) following the shorter delay in Qa-1 expression (A-Test score = 0.704). Conversely, simulations employing an increased delay to Qa-1 expression (82 hours) demonstrate a large, and scientifically significant, decrease in the peak population of CD8Treg cells with respect to control dynamics (A-Test score = 0.163). As would be expected, this demonstrates a large, and scientifically significant decrease between the two delay periods (A-Test score = 0.090). This indicates that the timing of Qa-1 expression by DCs has a significant affect on modulating the regulatory CD8Treg CTL response.

### Spatial competition between CD4Treg and CD8Treg cells around the dendritic cell modulates the regulatory response

**Figure 5:**
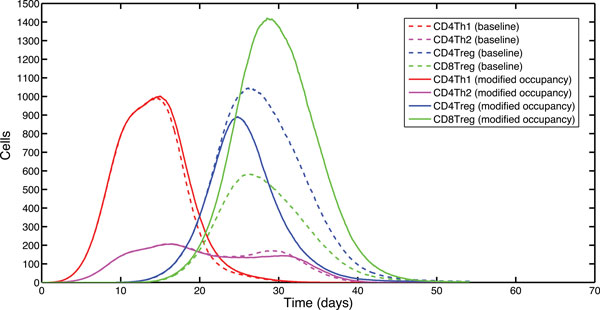
**Spatial dynamics of CD8Treg priming by DCs**. Median T cell effector populations in a sample of 1,000 simulator runs. The graph shows effector populations in two separate experiments: the solid curves are the results of running an experiment with modified CD8Treg occupancy rules, the dashed curves are the results from a baseline experiment.

Along with the temporal dynamics, the nature of *help *from CD4Treg cells within this system also imposes a spatial effect on CD8Treg priming. Simulations employing modified CD8Treg spatial occupancy rules, which allow CD8Treg cells to occupy space regardless of the CD4Treg that may already reside there (see Figure 5), demonstrate that the peak population of CD8Treg cells has undergone a large, scientifically significant, increase relative to the control simulation (A-Test score = 0.999). This indicates that spatial competition between CD4Treg and CD8Treg cells has a significant effect on modulating the regulatory CD8Treg CTL response.

## Conclusions

We have sought to investigate, through simulation, whether CD4Th1 and CD8Treg cells need to be primed on the same DC, and whether temporal and spatial mechanisms may regulate the CD8Treg CTL response within EAE. As Beeston et al [16] have firmly established that CD8Treg cells induce the killing of encephalitogenic CD4Th1 cells, our first hypothesis was that a significant degree of priming of these two cell populations occurs simultaneously around the same DCs. Observation of DC peptide presentation profiles has established that only 10% of DCs that are able to prime T cells simultaneously prime *both *CD4Th1 and CD8Treg populations. Furthermore, given our criteria for scientifically significant results, A-Test scores indicate that no significant difference in the ability of CD8Treg cells to regulate the CD4Th1 population arises from this manipulation. We therefore conclude that the killing of CD4Th1 cells as induced by CD8Treg cells does not critically rely on these T cell populations being primed on the same DCs, and conjecture that it is probable that killing of CD4Th1 cells does not take place around the DC, as such we reject the first hypothesis.

The second hypothesis concerned the timing of *help *provided by CD4Treg cells, through the time at which Qa-1 was expressed by DCs. We have found that delaying Qa-1 expression by DCs has a significant effect on the CD8Treg effector cell population. We therefore conclude that the timing of Qa-1 expression by DCs modulates the regulatory response, and as such accept the second *age of licensing *hypothesis, that the time delay in licensing is significant in modulating the regulatory CD8Treg CTL response. The third hypothesis concerned the binding space available around DCs, because although CD4Treg cells license DCs to provide critical *help *for the recovery from autoimmune response, their physical presence may limit the size of this CD8Treg CTL response. By relaxing spatial competition for CD8Treg, allowing them to occupy space and gain access to DCs regardless of CD4Treg presence, we found a significant effect on the CD8Treg effector cell population. We therefore conclude that spatial competition between CD4Treg and CD8Treg cells around the DC modulates the regulatory response, and as such accept the third *spatial saturation *hypothesis.

Results from the experiments regarding temporal dynamics and spatial dynamics suggest that both factors could serve to control the CTL response through altering the size of the CD8Treg cell population. Through this work, it is evident that although CD4Treg cells are required as part of the regulatory response via their capacity to provide *help*, they also exert a restraining influence on the population size of CD8Treg cells by virtue of their modulation of CD8Treg-DC interactions through spatial competition. Although these results are grounded specifically in the EAE system, we believe the spatial and temporal constraints that CD4Th *help *(provided by CD4Treg to CD8Treg in our simulation) imposes are applicable to CTL responses in general, and have significance in the design of treatments based on manipulating the CTL response. For example, by providing artificial *help *to DCs (through anti-CD40 antibodies) that prime cytotoxic T cells, the CD4Th cell-imposed delay in licensing can be removed, invoking an earlier CTL response of larger magnitude. If the CD4Th population were to be abrogated and suitable artificial cytokine and signalling surrogates administered, the spatial restrictions that *help *imposes, could be alleviated to effect a larger CTL response.

Taken together the analysis from these experiments show that CD4Th1 cells and Treg cells do not need to be primed by the same DC in order for down-regulation of the autoimmune response to occur. They have also shown that regulation of CTL responses (through CD8Treg cells within our simulation) appears to be limited by the nature of *help *provided by CD4Treg cells, and the spatial competition that results around priming dendritic cells. Although simulation results cannot, as yet, be confirmed using *in vitro *or *in vivo *testing, our work has shown that temporal dynamics of Qa-1 expression and spatial competition around DCs is very important for the modulation of the CTL regulatory response.

## Method

### Simulation runs

The simulator (ARTIMMUS) was previously developed [14] in Java and the MASON modelling and simulation framework [17]. Within simulation runs, immunization is through MBP, a myelin derivative, and adjuvant. CD4Th cells are initially naive and start without a polarisation; once polarised they make a probabilistic decision to become either type1 or type2. This is affected by the cytokine profiles present locally at the time of priming. The adjuvant produces a type1 polarisation in DCs that perceive them. Type 1 DCs secrete type1 cytokine, which faciltate the majority of CD4Th cells to adopt a type1 polarisation upon their activation, with a small residual number adopting a type2 polarisation. For the purposes of statistical analysis, 1000 individual simulations for each experiment were run on a server cluster. Matlab was used to analyze cell populations and population level behavior.

### Non-parametric A-tests

The ARTIMMUS simulator generates large volumes of data for each simulation run. Non-parametric effect magnitude tests [18,19], termed A-Tests, are used to measure scientific significance (effect size) of the difference between cell populations from simulation experiments with respect to control cell populations. The A-Test provides a score in the range [0.0, 1.0] that represents the probability that a randomly selected sample from population one is larger than a randomly selected sample from population two. Vargha and Delaney [19] provide thresholds to indicate the magnitude of difference between two populations. They indicate scores >0.71 and <0.29 to represent *large *differences, and we assume these boundaries to indicate scientific significance. For completeness, they also indicate: 0.64< score <0.71 and 0.29< score <0.36 to represent *medium *differences, and 0.50< score <0.56 and 0.44< score <0.50 to represent *small *differences. We have chosen to use a non-parametric test in order to avoid the assumption that the underlying populations of simulator results tend towards normality. We use an effect magnitude test to determine scientific significance in place of statistical significance because with computer simulations, a sufficiently large enough number of simulation runs can always be performed, which will reveal a statistical significance, unless the variable of interest truly has no effect. We use the A-Test in preference to simply measuring the difference in median values since the differences in medians does not take the spread of the data into account (for a more detailed justification please refer to Read et al [20]). The A-Test was used to assess the significance of experimental results with the control data.

### Mutually exclusive peptide presentation by dendritic cells

The significance of antigenic peptide presentation by DCs on the dynamics of CD8Treg priming was assessed. To investigate peptide presentation dynamics, we have manipulated ARTIMMUS such that presentation of antigenic peptide by DCs is *mutually exclusive*; DCs are no longer able to present both MBP- and TCR-derived peptides. This manipulation to ARTIMMUS allows DCs to efferocytose cells as normal, however only the peptide type (MBP- or TCR-derived) associated with the cell that was first engulfed and processed into functional peptides, can become presented on major histocompatibility complexes (MHCs) of the DC. This negates the ability of a single DC to prime both CD4Th1 and CD4Treg cells, allowing us to observe the effects on the system. To ensure individual DCs were only counted once, counts were taken of DCs that had entered apoptosis by the end of the simulation.

### Temporal dynamics of CD8Treg priming by dendritic cells

The significance of the effect of the time at which Qa-1 is expressed by DCs was assessed. Licensing of DCs to express Qa-1 within the *control *simulation code is via CD4Treg cells. In order to manipulate the time at which Qa-1 is expressed, we abrogated CD4Treg cells from the simulation, and to ensure continued priming of CD8Treg cells we incorporated functionality for expression of Qa-1 by DCs, following specific time delays. In this context, *timing of Qa-1 expression*, means the expression of optimal levels of Qa-1/TCR-peptide complexes (along with costimulatory molecules) on the surface of DCs enabling the priming of the CD8Treg.

A control experiment was performed to record the distribution of ages of DCs at their time of licensing. The overall median age of licensing was calculated as 74 hours. An investigation of variation in DC age at time of licensing showed that the overall age distribution could be divided into two distinct sub-populations. The median ages at the time of DC licensing for these two sub-populations were calculated as 60 hours and 82 hours [21]. These medians were used to parametrize enforced delays in the Qa-1 expression by DCs. The division between the two sub-populations related to DCs created before or after day 20. This is the point in the simulation (see Figure 2 Control Dynamics) where the number of CD4Treg cells and CD4Th1 cells are equal, with the CD4Treg cell population rapidly increasing and the CD4Th1 cell population rapidly decreasing.

### Spatial dynamics of CD8Treg priming by dendritic cells

In a preliminary experiment we recorded the number and nature of all T-cell neighbours of apoptotic DCs. From this data we were able to ascertain a median number of CD8Treg cells that were neighbours around a DC in a control experiment in which the CD4Treg population had been abrogated. We then used this number as an upper limit for the number of CD8Treg cells that could occupy a simulation grid space irrespective of how many CD4Treg cells were currently resident in that space. Using these modified CD8Treg occupancy rules, we ran further baseline simulations to examine the effect of reduced CD8Treg/CD4Treg spatial competition on CD8Treg cell population size.

## Competing interests

The authors declare that they have no competing interests.

## Authors' contributions

Williams undertook the mutually exclusive peptide presentation experimentation and was lead author on the development of the manuscript, Greaves performed the temporal dynamics and spatial dynamics experimentation and assisted in the manuscript production. Andrews, Read, Kumar and Timmis were involved in the design of the experiments and manuscript production. Kumar provided immunological interpretation of the results.

## Declarations

This article has been published as part of *BMC Bioinformatics *Volume 14 Supplement 6, 2013: Selected articles from the 10th International Conference on Artificial Immune Systems (ICARIS). The full contents of the supplement are available online at http://www.biomedcentral.com/bmcbioinformatics/supplements/14/S6.
